# Coronary Artery Disease and Prognosis of Heart Failure with Reduced Ejection Fraction

**DOI:** 10.3390/jcm12083028

**Published:** 2023-04-21

**Authors:** Lourdes Vicent, Jesús Álvarez-García, Rafael Vazquez-Garcia, José R. González-Juanatey, Miguel Rivera, Javier Segovia, Domingo Pascual-Figal, Ramón Bover, Fernando Worner, Francisco Fernández-Avilés, Albert Ariza-Sole, Manuel Martínez-Sellés

**Affiliations:** 1 Cardiology Department, Hospital Universitario 12 de Octubre, 28041 Madrid, Spainmmselles@secardiologia.es (M.M.-S.); 2 Cardiology Department, Hospital de la Santa Creu i Sant Pau, CIBERCV, 08025 Barcelona, Spain; 3 Cardiology Department, Puerta del Mar University Hospital, 11009 Cádiz, Spain; 4 Cardiology Department, Hospital Clínico Universitario de Santiago, CIBERCV, 15076 Santiago de Compostela, Spain; 5 Cardiology Department, University Hospital La Fe, 46026 Valencia, Spain; 6 Cardiology Department, Hospital Universitario Puerta de Hierro Majadahonda, CIBERCV, 28222 Madrid, Spain; 7 Cardiology Department, Hospital Virgen de la Arrixaca, Department of Medicine, University of Murcia, 30120 Murcia, Spain; 8 Centro Nacional de Investigaciones Cardiovasculares Carlos III (CNIC), 28029 Madrid, Spain; 9 Cardiology Department, Hospital Clínico San Carlos, 28040 Madrid, Spain; 10 Servicio de Cardiología, Hospital Universitari Arnau de Vilanova, 25198 Lleida, Spain; 11 Cardiology Department, Instituto de Investigación, Hospital General Universitario Gregorio Marañón, CIBERCV, 28007 Madrid, Spain; 12 Cardiology Department, Bellvitge University Hospital General, L’Hospitalet de Llobregat, 08907 Barcelona, Spain; 13 Facultad de Medicina, Universidad Complutense, 28040 Madrid, Spain; 14 Facultad de Medicina, Universidad Europea, 28670 Madrid, Spain

**Keywords:** heart failure, dilated cardiomyopathy, mortality, readmissions, ischemic heart disease, coronary artery disease

## Abstract

Our aim was to determine the prognostic impact of coronary artery disease (CAD) on heart failure with reduced ejection fraction (HFrEF) mortality and readmissions. From a prospective multicenter registry that included 1831 patients hospitalized due to heart failure, 583 had a left ventricular ejection fraction of <40%. In total, 266 patients (45.6%) had coronary artery disease as main etiology and 137 (23.5%) had idiopathic dilated cardiomyopathy (DCM), and they are the focus of this study. Significant differences were found in Charlson index (CAD 4.4 ± 2.8, idiopathic DCM 2.9 ± 2.4, *p* < 0.001), and in the number of previous hospitalizations (1.1 ± 1, 0.8 ± 1.2, respectively, *p* = 0.015). One-year mortality was similar in the two groups: idiopathic DCM (hazard ratio [HR] = 1), CAD (HR 1.50; 95% CI 0.83–2.70, *p* = 0.182). Mortality/readmissions were also comparable: CAD (HR 0.96; 95% CI 0.64–1.41, *p* = 0.81). Patients with idiopathic DCM had a higher probability of receiving a heart transplant than those with CAD (HR 4.6; 95% CI 1.4–13.4, *p* = 0.012). The prognosis of HFrEF is similar in patients with CAD etiology and in those with idiopathic DCM. Patients with idiopathic DCM were more prone to receive heart transplant.

## 1. Introduction

Heart failure (HF) is one of the leading causes of mortality and hospital admissions worldwide [1]. The etiology of HF with reduced left ventricular ejection fraction (HFrEF) is diverse [2]; however, the two main causes are coronary artery disease (CAD) and idiopathic dilated cardiomyopathies (DCM) [3,4]. The prognostic impact of HFrEF etiology is still under debate. Previous studies have established a worse prognosis in patients with CAD compared to other etiologies, such as hypertension or DCM [5,6,7]. However, a subanalysis of PARADIGM-HF (Prospective Comparison of ARNI [Angiotensin Receptor–Neprilysin Inhibitor] with ACEI [Angiotensin-Converting Enzyme Inhibitor] to Determine Impact on Global Mortality and Morbidity in Heart Failure Trial) found that patients with CAD had a higher crude incidence of mortality and readmissions; however, outcome did not differ by etiology (CAD, idiopathic, or hypertensive) when adjusting for other variables, such as age and comorbidity [8]. Moreover, most studies addressing the prognostic implications of HF etiology were performed before novel drugs development and cardiac devices [2,3,9,10]. Specifically, some of these improvements, such as implanted cardioverter defibrillators, are particularly effective in patients with CAD [11]. Ultimately, the clinical profile of HFrEF patients with idiopathic DCM compared to patients with CAD is usually different [8], and this fact might confound the real prognostic effect of etiology.

So, our objective was to assess the prognostic impact of CAD on mortality and hospitalizations in a large group of patients admitted with HFrEF in real-world practice. 

## 2. Methods

### 2.1. Study Population

This is a subanalysis of the Spanish Network for the Study of Heart Failure II registry (REDINSCOR II). The methodology of the study has been previously detailed [12,13]. Briefly, the REDINSCOR II is a prospective, multicenter, nationwide study including adults admitted for acute HF in 20 Spanish hospitals, from October 2013 to December 2014. All patients had a diagnosis of acute HF at admission, according to the definition of the current clinical practice guidelines [14] and, for this analysis, we only included those patients with HFrEF (left ventricular ejection fraction <40%) (n = 583). Patients received optimal HF therapy according to current guidelines in the recruitment years. Study data were comprehensively recorded, and quality controls took place regularly. The recorded variables include: (1) demographic data, such as previous medical history; (2) physical examinations for signs and symptoms; (3) complementary examinations (ECG, chest X ray, laboratory tests, echocardiography, coronary angiography, etc.); (4) detailed medical treatments and invasive procedures (mechanical circulatory support, mechanical ventilation, etc.). Follow-up data were obtained by telephone contact at 1, 3, 6, and 12 months after discharge.

This study was conducted in line with the Declaration of Helsinki and was approved by the Ethics Committee of the recruiting hospitals (9/12/2013 CEIC: 57/2013; 19/09/2013 CEIC: 13/2013). All patients provided written informed consent.

### 2.2. Study Variables and Outcomes

Patients were stratified into two groups according to HF etiology (CAD, idiopathic DCM). Patients with CAD etiology had to present a coronary artery disease of sufficient severity and extension to justify myocardial damage [3,15]. Idiopathic DCM was defined as a ventricular dilation, accompanied by deterioration of contractile function in the absence of an evident cause [10,16]. 

The primary endpoint was a composite of all-cause mortality, hospital readmissions, sudden cardiac death, or heart transplantation at 12 months. The secondary endpoint was mortality due to refractory HF. The combined endpoint hospital readmissions and mortality at 1 and 6 months was also assessed.

### 2.3. Statistical Analysis

Continuous variables are shown as mean (standard deviation) or median (interquartile interval) for non-normally distributed variables. Categorical data are presented as frequencies and percentages. Continuous quantitative variables were compared using Student’s t test and ANOVA for the comparison of means or the Wilcoxon rank sum in nonparametric data. Categorical variables were analyzed using the χ2 test and the Fischer exact test. Bonferroni’s correction was applied for multiple comparisons. 

Multivariate analysis included multiple logistic regression techniques and Cox regression modeling for the study endpoints. To determine which variables were entered into the final model, we used a sequential inclusion and exclusion method, with an inclusion *p* threshold lower than 0.05 and exclusion over than 0.1. The final model included age, previous heart failure admissions, diabetes, Charlson Comorbidity Index, glomerular filtration rate, HF therapies, rhythm, and anemia at discharge. All analyses were performed with the STATA software (version 14.0).

## 3. Results

### 3.1. Clinical Characteristics of the Study Population According to HF Etiology

From the 1831 patients enrolled in the registry, 583 had HFrEF and are the focus of our study. Mean age was 68.2 ± 12.8 years and 136 (23.3%) were women. In total, 266 patients (45.6%) had CAD as main etiology, and 137 (23.5%) had idiopathic DCM. Baseline demographic, clinical characteristics, and chronic treatments are shown in Table 1. Compared with CAD, patients with idiopathic DCM were younger, had a lower left ventricular ejection fraction, and were more frequently carriers of implanted cardioverter defibrillators. Compared with patients with CAD, they also had less comorbidity, a shorter time since the diagnosis, and a lower prescription of HF drugs before admission. In total, 118 CAD patients (44.4%) had a history of percutaneous coronary revascularization, 55 (20.8%) had undergone coronary artery bypass graft, and 33 (12.5%) had a history of both percutaneous and surgical coronary revascularization. In 60 patients (22.3%), CAD was considered diffuse and non-amenable to revascularization.

### 3.2. Management

Treatments administered during hospitalization and at hospital discharge are shown in Table 2. Compared with the CAD group, patients with idiopathic DCM were treated more frequently with a left ventricular assist device. Nitrates were mainly prescribed in patients with CAD. Patients with idiopathic DCM received beta blockers and mineralocorticoid receptor antagonists more frequently. There were no other relevant differences.

### 3.3. Prognostic Impact of CAD

**Figure 1:**
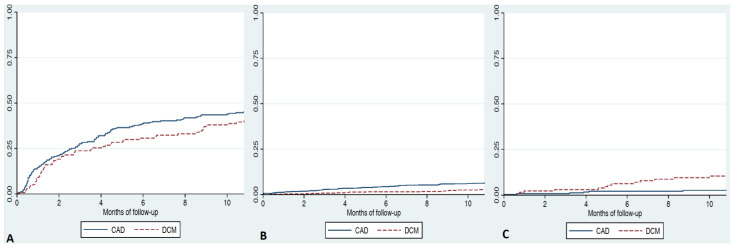
Adjusted Kaplan–Meier curves in patients admitted due to heart failure divided in three groups: idiopathic dilated cardiomyopathy (DCM), coronary artery disease (CAD)-ischemic. (**A**) Combined endpoint of death/heart failure readmission. (**B**) All-cause death. (**C**) Heart transplantation.

Events during follow-up are shown in Table 3. Independent predictors of 12-month mortality and readmissions are shown in Table 4. Patients with ischemic HF presented the highest 12-month mortality (24%); however, HF etiology was not independently associated with prognosis. The 1-year mortality was similar in patients with CAD (hazard ratio [HR] 1.50; 95% confidence interval [CI] 0.83–2.70, *p* = 0.182) compared to idiopathic DCM. Mortality/readmissions were also comparable in CAD etiology (HR 0.96; 95% CI 0.64–1.41, *p* = 0.81) compared to idiopathic DCM. Figure 1 shows Kaplan–Meier survival curves in the two groups. Patients with idiopathic DCM had a higher likelihood of receiving a heart transplant: HR 4.6 (95% CI 1.4–13.4, *p* = 0.012) compared to CAD patients.

## 4. Discussion

In our non-selected population of patients admitted to cardiology departments due to HFrEF, we have found that, after adjusting for comorbidities, patients with CAD etiology had a prognosis similar to idiopathic DCM with comparable adjusted mortality and readmissions.

Previous studies have found that the prevalence of nonischemic DCM ranges from 30 to 50% HF patients [16,17,18]. Patients with idiopathic DCM were younger, and had less cardiovascular risk factors and comorbidities than those with CAD. This is in agreement with preceding studies [3,8,9,10,19]. However, a better outcome in patients with idiopathic DCM compared to CAD etiology was not found in some previous studies [20,21,22,23]. A possible explanation is that previous reports have been obtained from clinical trials performed in the 1980s [20,21,24], and in patients with a recent myocardial infarction [20,25]. Moreover, the substantial differences in age and clinical characteristics between the two groups may explain, at least in part, this prognostic difference. In the Revascularization for Ischemic Ventricular Dysfunction (REVIVED) trial, myocardial revascularization in patients with ischemic left ventricular dysfunction was not associated with a reduction in mortality or hospitalizations [26]; therefore, other factors, beyond ischemia per se, would have a greater weight in the evolution of HF patients. The Surgical Treatment for Ischemic Heart Failure (STICH) trial and its extension study (STICHES) showed that the presence of viability in patients with ischemic cardiomyopathy had no impact on the long-term prognosis, and bypass surgery seems to have no short-term survival benefit [27]. The results of the 10-year follow-up suggested a long-term benefit of coronary artery bypass grafting and of surgical ventricular reconstruction. However, in the group of surgical ventricular reconstruction, there was no difference with and without coronary artery bypass grafting. In addition, patients included in the STICH trial had a mean age below 60 years, and younger patients are the ones that had the greatest reductions in mortality. Comorbid conditions are strongly associated with an adverse prognosis in HF [28]. In our registry, comorbidities were more frequently found in patients with CAD than in those with idiopathic DCM. For instance, anemia [29] and diabetes mellitus were more common in CAD than in patients with ICM, and were associated with a substantial increase in mortality and hospitalizations. After adjusting for comorbidities, we found no independent relation between CAD and prognosis. The prevalence of mitral regurgitation and chronic kidney disease tended to be more frequent among patients with CAD but were not independently associated with outcomes in multivariate analysis. 

Interestingly, we found that, compared with DCM, patients with CAD had a longer time of HF evolution since the diagnosis, a fact that may have also had an impact on outcomes. They also had a higher prescription of HF drugs before admission, in accordance with a more long-standing and advanced disease. Patients with CAD had a lower prescription of beta-blockers, possibly due to the greater comorbidity, including peripheral arterial disease and chronic obstructive pulmonary disease. However, at hospital discharge, the proportion of patients treated with these drugs was close to 90%. Previous HF hospital admissions are associated with increased mortality, readmissions, and adverse outcomes [30,31,32]. Patients with ischemic cardiomyopathy had a higher rate of previous hospital admissions. Regarding smoking and alcohol abuse, it should be noted that patients with ischemic cardiomyopathy had a longer time since the diagnosis, so many patients may have changed their unhealthy behavior. In fact, the highest proportion of former smokers was seen in patients with ischemic cardiomyopathy. 

Unexpectedly, the proportion of implantable cardioverter defibrillator carriers was higher in idiopathic DCM compared to CAD. Although there is more robust data on the effect of implantable cardioverter defibrillators in HFrEF patients with CAD [33,34], in our study, patients with idiopathic DCM were younger and had less comorbidity than those with CAD, and advanced age and comorbidities were associated with low rates of implantable cardioverter defibrillator use [33]. 

More than 10% of our patients with idiopathic DCM received a heart transplant during the year following the index hospital admission, a rate 4 times higher than in patients with CAD. Although in recent series, CAD is a major cause of heart transplant [35], CAD patients are older and frequently present comorbidities that contraindicate heart transplant [36]. Moreover, idiopathic DCM is associated with a better survival after heart transplant [37]. 

Our study has some limitations. Idiopathic DCM is a heterogeneous condition that may include undetected causes, such as viral infections/myocarditis, autoimmune disorders, unknown drugs toxicity, nutritional deficiencies, genetic/familiar cases, or infiltrative diseases [18]. This multicenter registry only included patients admitted to cardiology departments, and the clinical profile may differ from other patients admitted to other departments. Our study was performed before the advent of new HF drugs, such as sacubitril/valsartan and sodium–glucose co-transporter-2 inhibitors. Genetic testing was not addressed, and it could have had an important impact on idiopathic DCM prognosis. Moderate–severe mitral regurgitation is a prognostic factor in HF with reduced ejection fraction, but we have no information regarding the surgical or invasive treatment performed after the index hospital admission. Information regarding active ischemia in the CAD group was not available as a study variable, so we cannot assess its impact on the outcome of this group of patients. Information regarding medical treatment during follow-up was not available. Data regarding the number of hospital readmissions during follow-up according to etiology was not recorded. Despite the high number of patients enrolled, the specific subgroups may have included a relatively low number of patients to assess the natural history of HFrEF. Finally, follow-up duration was 12 months, and a longer follow-up period may have shown significant differences in outcomes according to HF etiology. This study is based on a large-scale national registry, and patient follow-up data are only available 12 months after inclusion in the study. Future studies are desirable in order to address the potential differences according to etiology at subclinical and earlier stages of the disease.

## 5. Conclusions

After an HFrEF admission, prognosis is similar in patients with CAD and in idiopathic DCM. Patients with idiopathic DCM were more prone to receive heart transplant.

## Figures and Tables

**Table 1:** Baseline demographic and clinical characteristics.

	Idiopathic DCM (n = 137)	CAD (n = 266)	*p*
**Age**	**63.6 ± 13.8**	**71.1 ± 11.1**	**<0.001**
**Female sex**	**33 (24.1)**	**52 (19.6)**	**0.10**
**Tobacco** - **Former smoker** - **Active smoker**	**29 (21.2)** **57 (41.6)**	**128 (48.3)** **44 (16.6)**	**0.01**
**Alcohol**	**31 (22.6)**	**30 (11.3)**	**0.003**
**Hypertension**	**76 (55.5)**	**224 (84.2)**	**<0.001**
**Diabetes**	**51 (37.2)**	**153 (57.5)**	**<0.001**
**Chronic kidney disease**	**29 (21.5)**	**100 (37.6)**	**<0.001**
Chronic obstructive pulmonary disease	25 (18.3)	41 (15.4)	0.71
Stroke	11 (8.0)	32 (12.0)	0.23
**Peripheral arterial disease**	**11 (8.0)**	**45 (16.9)**	**0.040**
**Anemia**	**35 (29.7)**	**146 (64.0)**	**<0.001**
**Charlson Comorbidity Index**	**2.9 ± 2.4**	**4.4 ± 2.8**	**<0.001**
Barthel Index for Activities of Daily Living	94 ± 16	92 ± 17	0.18
**Previous HF diagnosis**	**72 (52.6)**	**184 (69.2)**	**<0.001**
**Previous HF admissions**	**50 (37.8)**	**129 (48.7)**	**0.04**
**Number of previous HF admissions**	**0.8 ± 1.2**	**1.1 ± 1.8**	**0.01**
**Years since initial diagnosis**	**3.5 ± 5.4**	**4.2 ± 5.9**	**0.02**
**Angiotensin-converting enzyme inhibitors/Angiotensin II receptor blockers**	**78 (56.9)**	**198 (74.4)**	**<0.001**
**Betablockers**	**80 (58.4)**	**191 (71.8)**	**<0.001**
Loop diuretics	78 (56.9)	179 (67.3)	0.07
Thiazides	13 (9.5)	23 (8.7)	0.17
**Mineralocorticoid receptor antagonists**	**49 (35.8)**	**108 (40.8)**	**0.001**
Digoxin	11 (8.0)	23 (8.7)	0.21
**Nitrates**	**6 (4.4)**	**86 (32.3)**	**<0.001**
Hydralazine	5 (3.7)	13 (4.9)	0.24
**Cardiac resynchronization therapy**	**12 (8.8)**	**14 (5.3)**	**0.02**
**Implantable cardioverter defibrillator**	**29 (21.3)**	**42 (15.9)**	**0.001**
Chronic anticoagulation	47 (34.3)	98 (37.0)	0.69
Atrial fibrillation/flutter	47 (35.3)	80 (31.3)	0.072
Left bundle branch block	40 (30.1)	63 (25.3)	0.25
**QRS duration (ms)**	**128 ± 33**	**129 ± 36**	**0.003**
**Left ventricular ejection fraction (%)**	**25.2 ± 7.3**	**28.0 ± 7.0**	**<0.001**
**Left ventricular end diastolic diameter (mm)**	**64.2 ± 9.2**	**62.6 ± 9.2**	**<0.001**
Moderate–severe mitral regurgitation	79 (60.8)	135 (53.1)	0.21
Systolic pulmonary artery pressure (mmHg)	47.0 ± 12.8	46.4 ± 13.5	0.72
Tricuspid annular plane systolic excursion (mm)	16.5 ± 4.6	16.2 ± 4.4	0.19
**Glomerular filtration rate (mL/min)**	**70.5 ± 32.2**	**61.5 ± 30.1**	**<0.001**
N-terminal-pro B-type natriuretic peptide	984 ± 1091	1231 ± 1596	0.35
Systolic blood pressure (mmHg)	110 ± 16	113 ± 16	0.27
Diastolic blood pressure (mmHg)	68 ± 12	66 ± 11	0.21
Heart rate (beats/min)	73 ± 14	73 ± 14	0.43

Data are shown as number of patients and percentages for categorical variables, and mean ± standard deviation for continuous variables. CAD: Coronary artery disease. DCM: Dilated cardiomyopathy. HF: Heart failure.

**Table 2:** Treatments during hospital admission and at hospital discharge.

	Idiopathic DCM (n = 137)	CAD (n = 266)	*p*
Non-invasive mechanical ventilation	4 (3.0)	15 (5.7)	0.15
Invasive mechanical ventilation	1 (0.8)	3 (1.1)	0.24
Mechanical circulatory support - Intra-aortic balloon pump - Left ventricular assist device	1 (0.8) 1 (0.8)	1 (0.4) 13 (5.1)	0.09 0.02
Angiotensin-converting enzyme inhibitors/Angiotensin II receptor blockers	113 (82.5)	192 (72.2)	0.06
**Betablockers**	**121 (91.0)**	**223 (87.5)**	**0.04**
Loop diuretics	123 (89.8)	229 (86.1)	0.72
Thiazides	8 (5.8)	14 (5.3)	0.69
**Mineralocorticoid receptor antagonists**	**103 (76.9)**	**150 (59.8)**	**0.001**
**Digoxin**	**26 (9.8)**	**26 (9.8)**	**<0.001**
**Nitrates**	**11 (8.0)**	**67 (25.2)**	**<0.001**
Ivabradine	23 (16.8)	40 (15.0)	0.08
Hydralazine	5 (3.7)	10 (3.8)	0.67
Death during hospital admission	3 (2.2)	12 (4.5)	0.29
Length of hospital stay (days)	11.5 ± 12.4	12.0 ± 9.3	0.85

Data are shown as number of patients and percentages for categorical variables, and mean ± standard deviation for continuous variables. CAD: Coronary artery disease. DCM: Dilated cardiomyopathy.

**Table 3:** Events during follow-up and independent predictors of 12-month mortality and readmissions.

	Idiopathic DCM (n = 137)	CAD (n = 266)	*p*
Hospital readmissions			
1 month	12 (8.9)	38 (15.0)	0.366
6 months	75 (28.2)	36 (26.3)	0.388
12 months	88 (33.1)	44 (32.1)	0.845
Death during follow-up			
1 month	3 (2.2)	19 (7.1)	0.038
**6 months**	**11 (8.0)**	**46 (17.3)**	**0.008**
**12 months**	**18 (13.1)**	**63 (23.7)**	**0.010**
**Heart transplant at 12 months**	**15 (11.0)**	**10 (3.8)**	**<0.001**
**Death due to refractory HF at 12 months**	**9 (6.7)**	**35 (13.1)**	**0.037**
**Death due to cardiovascular causes at 12 months**	**11 (8.0)**	**44 (16.5)**	**0.04**
Sudden cardiac death at 12 months	2 (1.5)	9 (3.4)	0.24

Data are shown as number of patients and percentages for categorical variables, and mean ± standard deviation for continuous variables. CAD: Coronary artery disease. DCM: Dilated cardiomyopathy.

**Table 4:** Independent predictors of 12-month mortality and readmissions by Cox regression analysis.

**12-Month Mortality**	**HR (95% CI)**	***p***
**Previous HF admissions**	**1.23 (1.70–1.41)**	**<0.001**
**Diabetes mellitus**	**1.21 (1.05–1.38)**	**0.008**
**Angiotensin-converting enzyme inhibitors/Angiotensin II receptor blockers**	**0.44 (0.26–0.75)**	**0.002**
CAD *	1.50 (0.83–2.70)	0.182
**Anemia**	**2.20 (1.22–3.01)**	**<0.001**
**12-month mortality/readmissions**	**HR (95% CI)**	**p**
**Previous HF admissions**	**1.32 (1.189– 1.47)**	**<0.001**
**Diabetes mellitus**	**1.14 (1.02–1.41)**	**0.04**
**Angiotensin-converting enzyme inhibitors/Angiotensin II receptor blockers**	**0.61 (0.41–0.88)**	**0.009**
CAD *	0.96 (0.64–1.41)	0.81
**Anemia**	**1.73 (1.26–2.35)**	**0.001**
**12-month heart transplantation**	**HR (95% CI)**	**p**
**Older age**	**0.92 (0.89–0.96)**	**<0.001**
**Previous HF admissions**	**1.64 (1.21–2.22)**	**0.002**
**Betablockers**	**0.35 (0.10–0.89)**	**0.008**
Idiopathic DCM *	**4.6 (1.40–13.4)**	**0.012**
**Hemoglobin at discharge**	**0.97 (0.94–0.99)**	**0.029**

* Idiopathic DCM as reference. Ischemic DCM as reference. CI: Confidence interval; HR: hazard ratio. CAD: Coronary artery disease. DCM: Dilated cardiomyopathy. HF: Heart failure.

## Data Availability

The data will be shared on reasonable request to the corresponding author (with additional anonymization to avoid patient identification).

## Abbreviations

DCM	Dilated cardiomyopathy
HF	Heart failure
